# Effects of Diode Laser, Gaseous Ozone, and Medical Dressings on* Enterococcus faecalis* Biofilms in the Root Canal Ex Vivo

**DOI:** 10.1155/2017/6321850

**Published:** 2017-04-10

**Authors:** Kerstin Bitter, Alexander Vlassakidis, Mediha Niepel, Daniela Hoedke, Julia Schulze, Konrad Neumann, Annette Moter, Jörn Noetzel

**Affiliations:** ^1^Department of Operative Dentistry and Preventive Dentistry, CharitéCentrum 3, Charité-Universitätsmedizin Berlin, Aßmannshauser Str. 4-6, 14197 Berlin, Germany; ^2^Department of Periodontology and Synoptic Dentistry, CharitéCentrum 3, Charité-Universitätsmedizin Berlin, Aßmannshauser Str. 4-6, 14197 Berlin, Germany; ^3^Institute of Microbiology and Hygiene, CharitéCentrum 5, Charité-Universitätsmedizin Berlin, Sylter Straße 2, 13353 Berlin, Germany; ^4^Department of Medical Informatics, Biometry and Epidemiology, CharitéCentrum 4, Charité-Universitätsmedizin Berlin, Hindenburgdamm 30, 12203 Berlin, Germany; ^5^Biofilmcenter, German Heart Institute, Deutsches Herzzentrum Berlin (DHZB), Hindenburgdamm 30, 12203 Berlin, Germany; ^6^Private Practice Mutlangen, Schillerstraße 5, 73557 Mutlangen, Germany

## Abstract

The objective was to compare the antibacterial effects of adjunctive disinfection using diode laser and gaseous ozone compared to the medical dressings calcium hydroxide (Ca(OH)_2_) and chlorhexidine gel (CHX-Gel) on* Enterococcus faecalis* biofilms in human root canals ex vivo. Root canals of 180 human extracted teeth were infected by* E. faecalis* and divided into 3 main groups (G): G1, control; G2, instrumentation and irrigation using 0.9% NaCl; G3, instrumentation and irrigation using 1% NaOCl. In each main group, the following treatments were applied: gaseous ozone, diode laser, and medical dressings of Ca(OH)_2_ or CHX-Gel for 7 days (*n* = 15). Reduction of colony forming units (CFUs) inside the root canal of planktons and frequencies of adherent bacteria after treatment were calculated. Bacterial reduction was significantly affected by the irrigation protocol (*p* < 0.0005) and the disinfection method (*p* < 0.0005), and a significant interaction between both factors could be observed (*p* < 0.0005; ANOVA). In G3 (instrumentation using 1% NaOCl), no significant effect of disinfection methods could be demonstrated on planktonic bacteria (*p* = 0.062; ANOVA) and frequencies of adherent bacteria (*p* > 0.05; chi-square test). Instrumentation and irrigation using NaOCl combined with ozone or laser application resulted in comparable bacterial reduction on* E. faecalis* to the application of medical dressings.

## 1. Introduction

The control of an endodontic infection is affected by the following factors: host defense, instrumentation and irrigation of the root canal system, locally used intracanal medicaments between appointments, the root canal filling, and the coronal restoration [1].* E. faecalis* has been described as the most frequent species found in retreatment cases with a prevalence of up to 90% [2, 3]. One major key element of successful one- or multiple-visit root canal treatment is the chemomechanical debridement of the root canal including instrumentation and irrigation using antimicrobial solutions [4]. Anatomical complexities of the root canal system as well as the recalcitrance of microbial biofilms often demonstrate a serious challenge to effective root canal disinfection [5, 6]. Therefore, in the treatment of apical periodontitis, intracanal medication has been recommended to eliminate bacteria from the root canal system that survived instrumentation and irrigation [7]. The intracanal medicament calcium hydroxide (Ca(OH)_2_) is strongly alkaline and dissociates into calcium and hydroxide ions in aqueous solution resulting in an antibacterial effect and a tissue-dissolving capacity; however, the antimicrobial activity seems to depend on the direct contact of Ca(OH)_2_ with the bacteria [8, 9]. Because of its low solubility and diffusibility, Ca(OH)_2_ reveals a reduced effect against bacteria located in pulpal remnants, crevices, and isthmi in the canal system and the dentinal tubules especially against* E. faecalis* [10]. Moreover, complete removal of Ca(OH)_2_ from the root canal system irrespective of the irrigation solution or system is difficult to achieve because of the complexity of its anatomy [11–13]. Remnants of Ca(OH)_2_ may impair the sealing ability of the root canal filling [14, 15] and therefore alternative options concerning further intracanal medicaments or disinfection methods are of interest.

Chlorhexidine (CHX) is a synthetic cationic bisguanide that is positively charged. The hydrophobic and lipophilic molecule interacts with phospholipids and lipopolysaccharides on the cell membrane of bacteria and is able to enter the bacterial cells through active or passive transport mechanisms [16]. A randomized clinical trial analyzed the antibacterial effectiveness of the intracanal medicaments Ca(OH)_2_ and 2% CHX-Gel in teeth with chronic apical periodontitis and revealed a comparable effect [17]. Data of ex vivo studies demonstrated that 2% CHX-Gel as an intracanal medicament was more effective against* E. faecalis* compared to Ca(OH)_2_ [18, 19]. Nevertheless, the antimicrobial activity of CHX gel is affected by the time it remains inside the root canal because it is not able to act as a physical barrier [20]. Both intracanal medicaments require a second appointment to remove the medicament; consequently, a single-visit approach is not possible and complete removal of the medicament from the root canal system remains questionable. Moreover, reinfection of the root canal system is possible to occur during appointments. In addition, recent clinical data and meta-analyses demonstrated no significant differences of success rate and postoperative pain of single-visit or multiple-visit endodontic treatment [21–24]. However, during one-visit root canal treatment, adequate disinfection of the whole root canal system has to be ensured within one session.

Further disinfection methods besides the application of intracanal medicaments and irrigation solutions have been suggested to enhance the removal of residual bacteria from the root canal system. Ozone (O_3_) is a naturally occurring gas and is an energized, unstable form of oxygen that readily dissociates back into oxygen (O_2_) and singlet oxygen (O_1_), which is a reactive form of oxygen and is capable of oxidizing cells. It is able to destroy biomolecules and cell walls of bacteria [25]. Ozone gas (HealOzone; KaVo, Biberach, Germany) as an adjunctive disinfection method has been suggested to be used clinically in endodontic treatment but the results of studies on its efficacy against endodontic pathogens have been inconsistent [26]. Questions remain about the optimum duration and concentration of ozone gas that should be used [27]. Ozone demonstrated an antibacterial effect on planktonic* E. faecalis* cells but revealed a little effect on cells embedded in a biofilm structure [27] and was not comparable with the antibacterial effect of sodium hypochlorite [27–30]. In contrast to that, another study demonstrated that gaseous and aqueous ozone were as effective as NaOCl and CHX being able to completely remove the bacterial biofilm inside the root canal ex vivo [31].

The physical effect of laser (Light Amplification by Stimulated Emission of Radiation) is based on producing a light beam with high energy density through induced emission of atoms in the laser medium. The physical interaction between laser and tissue is determined by the adsorption spectrum of the tissue. Provided that the wavelength of the laser corresponds to the adsorption spectrum of the tissue, a linear biological effect characterized by hyperthermia (37–60°C), coagulation (60–100°C), carbonization (100–400°C), and evaporation (>400°C) on tissue cells is induced [32]. The application of diode laser irradiation has been suggested as an effective adjunctive antibacterial disinfectant in the root canal [33]. The antibacterial effect of diode laser irradiation has been attributed to its greater depth of penetration up to 1000 *μ*m into the dentinal tubules when compared to the penetration depth of chemical disinfectants, which were limited to 100 *μ*m in a recent in vitro study [34]. Accordingly, Gutknecht et al. demonstrated that diode laser with 980 nm wavelength can eliminate* E. faecalis* up to a penetration depth of 500 *μ*m effectively [35].

Little is known about the combination of irrigation protocols and adjunctive disinfection methods possibly enabling a one-visit endodontic treatment in comparison to the conventional method of using intracanal medicaments in a multiple-visit endodontic treatment. Consequently, the aim of the present study was to analyze the antimicrobial efficacy of gaseous ozone and diode laser in combination with various irrigation protocols in comparison to the application of intracanal medicaments (Ca(OH)_2_ and CHX-Gel) against* E. faecalis* biofilms in root canals of extracted human front teeth ex vivo.

The null hypothesis of the present study was that no difference in bacterial reduction between the disinfection methods and the intracanal medicaments in combination with the irrigation protocols would exist inside the root canal lumen (planktonic bacteria) and in the root canal dentin (adherent bacteria).

## 2. Methods

### 2.1. Sample Preparation

180 extracted, intact, human, upper canines with a single canal without distinct curvature were obtained with written informed consent under an ethics-approved protocol (EA4/102/14) by the Ethical Review Committee of the Charité-Universitätsmedizin Berlin, Germany, and cleaned with ultrasonic scalers (SONICFlex; KaVo, Biberach, Germany). Crowns were removed, all roots were shortened to 19.5 mm, and all samples were sterilized using ethylene dioxide (Campus Benjamin Franklin, Charité-Universitätsmedizin Berlin, Berlin, Germany).

**Figure 1:**
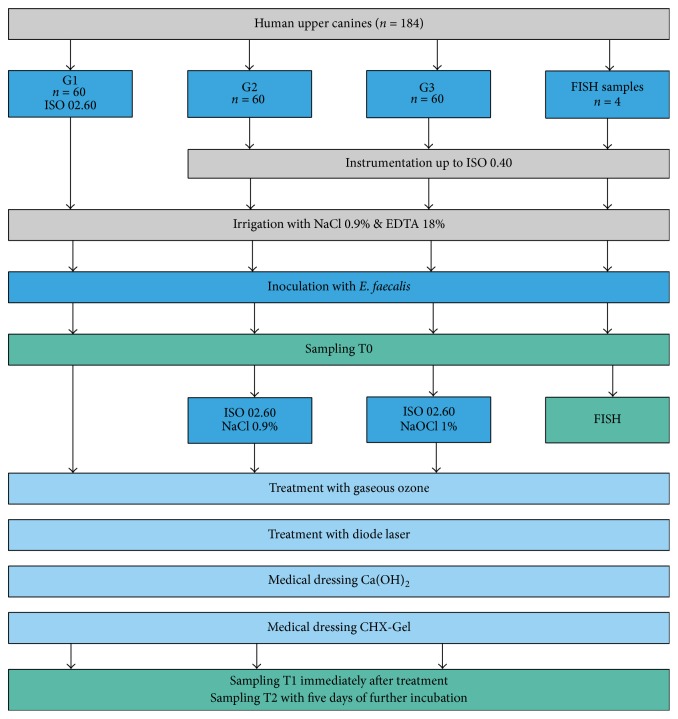
Flow chart illustrating study design and experimental procedure.

Subsequently, all teeth were randomly divided into three groups (G1–G3, *n* = 60). The coronal portion of the root canals was enlarged using Gates Glidden burs size 6 to 4. In G1 root canal enlargement was performed up to size 60 with 0.20 taper, whereas instrumentation limited to size 40, 0.20 taper, was carried out in G2 and G3 using Flexmaster rotary files (VDW, Munich, Germany). Irrigation was performed using sterile sodium chloride (0.9% NaCl, pharmacy of Charité-Universitätsmedizin Berlin, Germany). After initial root canal instrumentation, the smear layer was removed in all samples using 18% ethylenediaminetetraacetic acid (EDTA 18% Solution, Ultradent Products Inc., South Jordan, Utah, USA). After covering the root surfaces with nail varnish (Lilliput Nagellack, Kron 1959; Wiesbaden, Germany), each tooth was embedded into closable cryotubes (Carl Roth, Karlsruhe, Germany) using epoxy resin (Technovit 4071; Heraeus Kulzer, Hanau, Germany). Subsequently, all teeth were sterilized once again. Prior to inoculation of* E. faecalis*, sterility was tested by storing the teeth in sterile boxes (50 mL Falcon tubes; Sarstedt, Numbrecht, Germany) with sterile brain-heart-bouillon (BHI; SIFRIN, Berlin, Germany) at 37°C under anaerobic conditions for seven days. Clear bouillon after seven days indicated sterility. The whole study design is illustrated in Figure 1.

### 2.2. Inoculation of* E. faecalis*

Following sterilization, the root canals were infected with a suspension of 30 *μ*L* E. faecalis* (ATCC 29212) (optical density 0.1) in Tryptic Soy Broth (TSB, Sigma-Aldrich, St. Louis, MO, USA) with 0.25% glucose. After 24 h of incubation at 37°C, the root canals were infected once again according to the procedure described above. The biofilm was incubated for six days at 37°C in CO_2_ atmosphere with daily addition of sterile TSB to ensure constant liquid levels in the root.

### 2.3. Root Canal Treatment

In G2 and G3 root canal enlargement to size 60, taper 0.20, was performed using sterile saline solution in G2 and sodium hypochlorite (1% NaOCl, pharmacy of Charité-Universitätsmedizin Berlin, Germany) in G3. During instrumentation, irrigation was performed using 2 mL irrigation solution after each change of file size and final irrigation using 3 mL irrigation solution in each group. All root canals were dried using paper points ISO 60 (paper point ISO 60; VDW, Munich, Germany).

Subsequently, the following disinfection protocols (A–D) were immediately applied.

(A) Application of gaseous ozone was carried out with a hand piece (HealOzone plus 2131C, KaVo) using sterile, disposable silicone caps (HealOzone Delivery Cup, 6 mm, KaVo) and endodontic cannulas (HealOzone application cannulas, 24 mm, KaVo). The cannulas were dropped into the root canals and gaseous ozone was applied twice for 60 s with a flow rate of 100 mL/min in each period (ozone concentration 2100 ppm which is equivalent to 4.49 g/m^3^).

(B) Diode laser application was executed via the endodontic program of the GENTLEray 980 Laser (KaVo, Biberach, Germany) with the following setting: 2.5 W at an average of 0.8 W, wavelength 980 nm. The glass fiber of the diode laser (Bare Fiber NIR Q 300 K, 200 *μ*m; Asclepion Laser Technologies, Jena, Germany) was dropped carefully into the root canals at working length −1 mm. The fiber was moved 4 times in a rotary manner along the dentin surface of each root canal wall in apical-coronal direction with a speed of 3 mm per second for 10 s.

(C and D) Medical dressings of Ca(OH)_2_ (UltraCal XS, Ultradent, South Jordan, Utah, USA) and CHX-Gel (Chlorhexamed 1% Gel, GlaxoSmithKline, London, UK) were applied into the root canal in an apical-coronal movement with sterile disposable cannula until the canal was completely filled and thereafter the samples were stored for 7 days at 37°C.

### 2.4. Sample Preparation for Microscopic Biofilm Evaluation

To confirm the establishment of biofilms in the root canals, four additional specimens were inoculated with* E. faecalis* as described above and fixated in 3.7% paraformaldehyde (3 vols.) in PBS (1 vol.) for 16 h at 4°C and then washed with sterile PBS and stored in a mixture of 100% ethanol and PBS (1 : 1). The root canals were filled using cold polymerizing resin (Technovit 8100; Heraeus Kulzer, Hanau, Germany) and the roots were also embedded using the same material. The roots were sectioned horizontally to the long axis of the root using a circular saw (Leitz 1600, Leitz GmbH & Co. KG, Oberkochen, Germany) and ground flat (grinding system Exakt, 400 CS, grinding paper P 1200, Exact, Apparatebau). Thereafter, unspecific DNA staining with a blue fluorescent dye (DAPI: 4′,6-diamidino-2-phenylindole dihydrochloride, Thermo Fisher, Waltham, USA) was performed. Imaging was performed using an epifluorescence microscope (Axioplan 2, Zeiss, Jena, Germany).

### 2.5. Sampling of Planktonic and Adherent Bacteria and Determination of Colony Forming Units

Sampling of bacteria was performed at three different time points (T0–T2): before treatment (T0), immediately after therapy (T1), and after further incubation (T2) of exemplary samples that revealed no bacterial count after T1. Sampling of planktonic bacteria from the liquid of each canal was determined by placing one sterile paper point size 40 (paper point ISO 40; VDW, Munich, Germany) into the root canal until it was soaked up with fluid up to the mark of 18 mm. Each paper point was placed into 1995 *μ*L sterile NaCl, vortexed for 30 s, and diluted serially before plating on culture plates (Columbia agar plates with 5% sheep blood; Heipha, Eppelheim, Germany).

Bacteria from dentin were taken by moving a Hedstroem file ISO size 60 three times along the dentin wall from apical to coronal position and placing the file into 1.995 mL NaCl in a cryotube. After vortexing for 30 s, the bacterial fluid was plated on culture plates.

All plates were incubated in CO_2_ atmosphere for 24 h at 37°C. The number of colony forming units (CFUs) was counted.

### 2.6. Statistical Evaluation

Kruskal-Wallis test was performed for comparison of baseline infection.

Before and after therapy, CFU counts of the planktonic bacteria were transformed in log_10_ scale and logarithmic reduction factors were calculated. Univariate variance analyses using logarithmic reduction factor as a dependent variable were carried out to determine the effect of irrigation protocol (factor 1) and of disinfection method (factor 2). Post hoc tests (Tukey's HSD) were performed to assess differences in the effects of different irrigation protocols and disinfection methods.

Categories of final bacterial counts were applied for paper point and dentin samples, respectively (1 < detection limit; 2 ≤ 47,500 CFUs/mL or ≤20,000 CFUs/mg; 3 > 47,500 CFUs/mL or >20,000 CFUs/mg). The distribution of all values for this classification was recorded in cross tabulations and chi-square tests (corrected *p* value, *p* = 0.0083).

All analyses were performed using IBM SPSS Statistics 22 (SPSS, IBM, Munich, Germany).

## 3. Results

### 3.1. Fluorescence Detection of* E. faecalis* Biofilms

**Figure 2:**
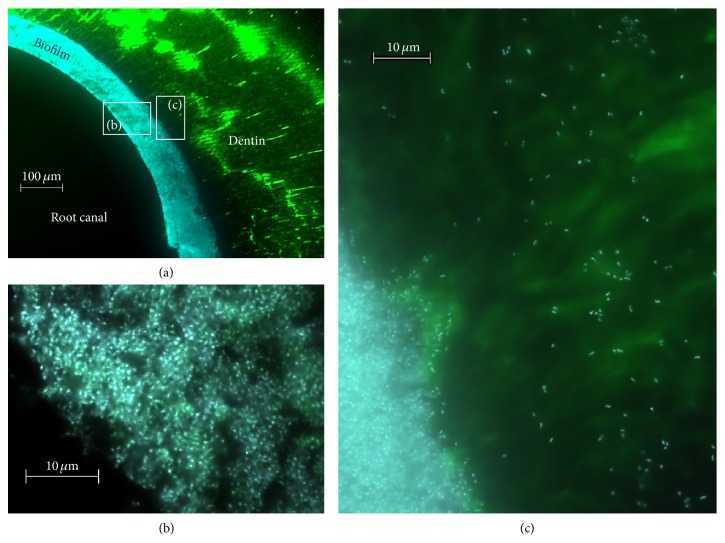
(a) Overview of a cross section of the root canal; the blue stained multilayered biofilm of* E. faecalis* is clearly visible; green background fluorescence of the root canal dentin. The white boxes indicate the areas that are displayed in (b) and (c). (b) Higher magnification of the multilayered biofilm located on the root canal dentin. (c) A mature biofilm has been formed inside the root canal and single bacterial cells are visible inside the dentinal tubules.

Figures 2(a)–2(c) reveal successful formation of a multilayered biofilm of* E. faecalis* in the root canal located on the root canal dentin in the root canal lumen as well as inside the dentinal tubules.

### 3.2. Quantitative Evaluation

The mean value of the initial bacterial count of all 180 samples was calculated at 2.57 × 10^6^ CFUs/mL (SD ± 2.62 × 10^6^). No significant differences between groups were detected at baseline (*p* = 0.057, Kruskal-Wallis test).

#### 3.2.1. CFUs of Planktonic Bacteria from the Root Canal Lumen

*Logarithmic* bacterial reduction was significantly affected by the irrigation protocol (*p* < 0.0005) and the disinfection method (*p* < 0.0005), and a significant interaction between both factors could be observed (*p* < 0.0005; ANOVA). Concerning the irrigation protocol, irrigation using 1% NaOCl revealed significantly higher bacterial reduction compared to G1 and G2 (*p* < 0.0005; Tukey's HSD). Disinfection using Ca(OH)_2_ revealed significantly higher bacterial reduction compared to all other methods (*p* ≤ 0.014), whereas laser application revealed significantly lower bacterial reduction compared to ozone treatment (*p* = 0.014) and the investigated medical dressings (*p* < 0.0005). The application of ozone and CHX-Gel did not differ with respect to bacterial reduction (*p* = 0.222; Tukey's HSD).

**Figure 3:**
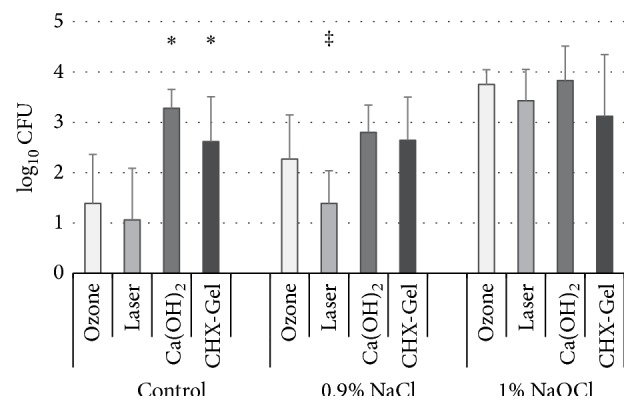
*Logarithmic* bacterial reduction of planktonic bacteria with respect to main groups 1–3 and the respective disinfection methods. In the control group without irrigation and instrumentation, intramedical dressing with Ca(OH)_2_ or CHX-Gel revealed significantly higher bacterial reduction compared to both ozone and laser treatment indicated by (^*∗*^*p* ≤ 0.001; Tukey's HSD). After irrigation using 0.9% NaCl, laser application resulted in a significant lower bacterial reduction compared to all other treatments indicated by (^‡^*p* ≤ 0.011; Tukey's HSD). No significant differences between groups could be observed when irrigation was performed with 1% NaOCl.

Analyses with respect to the applied irrigation protocol revealed for G1 (no further instrumentation) and G2 (instrumentation with NaCl) a significant effect of the disinfection method on bacterial reduction (*p* < 0.0005; ANOVA). For G1, application of the medical dressings revealed significantly higher bacterial reduction compared to laser or ozone treatment (*p* ≤ 0.001; Tukey's HSD). For main group G2 (instrumentation using NaCl), laser treatment revealed significantly lower bacterial reduction compared to all other disinfection methods (*p* ≤ 0.011; Tukey's HSD). In main group G3 (instrumentation using 1% NaOCl), no significant effect of the disinfection method could be demonstrated (*p* = 0.062; ANOVA) (Figure 3).

#### 3.2.2. Analyses of Adherent Bacteria

**Figure 4:**
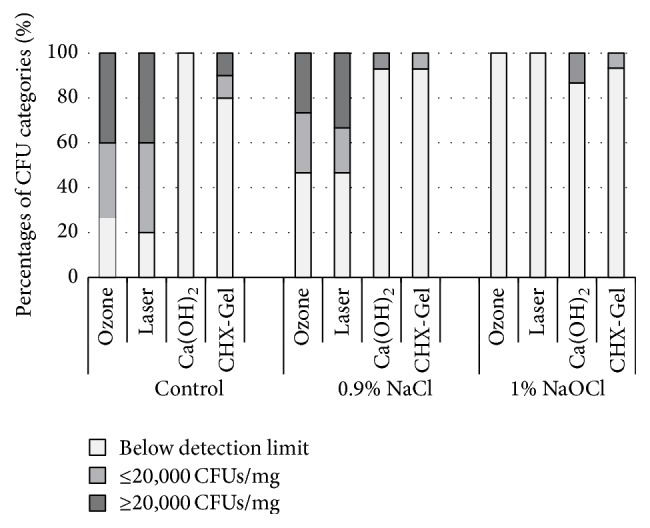
Percentile distribution of CFUs from adherent bacteria with respect to categories.

In G1, medical dressings using Ca(OH)_2_ or CHX-Gel revealed significantly lower categories of CFU counts compared to ozone and laser treatment (*p* ≤ 0.004; chi-square test), whereas the latter did not differ significantly (*p* = 1.000; chi-square test). In G2 and G3, no significant differences between groups could be detected (*p* > 0.0083; chi-square test) (Figure 4).

Exemplary dentin samples with CFU levels below detection level from each group were further incubated for 5 days and bacterial growth was evaluated. Significant differences between subgroups were detected (*p* < 0.0005; chi-square test). Medical dressings using CHX-Gel or Ca(OH)_2_ revealed in 85% and 52.6% of all incubated samples no further bacterial growth whereas ozone and laser treatment demonstrated bacterial regrowth in 78.6% and 81.8% of all samples.

## 4. Discussion

The present study analyzed the antimicrobial efficacy of gaseous ozone and diode laser application without further instrumentation and irrigation as well as in combination with an antibacterial irrigation protocol in comparison to the application of intracanal medicaments against* E. faecalis* biofilms in root canals of extracted human upper canines ex vivo.

The null hypothesis of the present study has to be partly rejected because significant differences with respect to the instrumentation and irrigation protocol as well as to the various disinfection methods could be detected. In combination with antibacterial irrigation using 1% NaOCl, no significant differences between the various disinfection methods could be observed.

The present ex vivo study employed a monospecies biofilm model inside the root canal of upper canines using* E. faecalis*. Upper canines exhibiting only one straight root canal with a standardized length of 19.5 mm were selected for the present study. After initial apical preparation up to ISO size 40, it can be assumed that uniform colonization of these root canals could be achieved.* E. faecalis* has been shown to be resistant against disinfecting agents and antibiotics [36] and can be effectively colonized; it forms a biofilm on root canal walls and invades dentinal tubules [37, 38]. Therefore, this monospecies biofilm model was used to reproduce the same biofilm-like structure in each of the investigated root canal samples with a species that is difficult to eliminate by chemomechanical debridement [36, 39]. Successful biofilm formation could be validated by fluorescence microscopic imaging where colonization of the root canal walls as well as penetration into the dentinal tubules (Figure 2) could be clearly visualized.

Nevertheless, one requirement for laboratory studies that aim to investigate the antimicrobial effects of various disinfection methods is to use models that closely resemble in vivo conditions [40]. Consequently, multispecies biofilm models for root canal disinfection ex vivo have been developed [41]. However, for in vitro testing reproducible infection of the root canals is important. For that reason, a monospecies biofilm model using* E. faecalis* was applied; moreover, front teeth with straight root canals for achieving comparable bacterial loads and standardized sampling were used. Additionally, sampling of planktonic and adherent bacteria was conducted and further incubation of exemplary samples that demonstrated no bacterial growth immediately after treatment was performed with the aim of detecting remnant bacteria that could only be detected after further growing.

The present study design confirmed the effect of chemomechanical debridement using 1% NaOCl compared to instrumentation of the root canal alone, as demonstrated previously [42]. NaOCl in concentrations of 1.0% and 5.0% has shown high antibacterial activity in a contact test [43], and residual NaOCl inside dentinal tubules has been regarded as crucial for effective disinfection [44]. In the present study, no blocking of NaOCl using sodium thiosulphate was performed, and consequently a continued effect of the applied disinfection protocol or a so-called carry-over effect inside the canal or the agar plate cannot be excluded [44]. Nevertheless, data on the carry-over effect of NaOCl are controversial and the effect seems to be negligible up to a NaOCl concentration of 3% [45, 46].

The present study design allows conclusions about the antimicrobial effects of the various disinfection methods with respect to instrumentation and irrigation protocol. Without further instrumentation of the root canal, the antibacterial effects of the investigated medical dressings were significantly higher compared to laser or ozone treatment alone.

For ozone treatment alone, these results have been corroborated in a recent ex vivo study where gaseous ozone treatment for 120 s resulted in 100% of samples with* E. faecalis* regrowth [47]. Additionally, it has been demonstrated previously that ozone had little antibacterial effects on* E. faecalis* cells embedded in a biofilm structure [27]. Conversely, Huth et al. achieved complete elimination of* E. faecalis* biofilms after application of gaseous ozone in a high concentration of 53 g/m^3^ for 1 min or lower concentrations with increased application time and concluded that the antibacterial effects of gaseous ozone were dose- and time-dependent [31]. The optimum duration of application and concentration of gaseous ozone are still a matter of debate and may lead to inconsistent results of its antibacterial efficacy [26]. Application time of gaseous ozone was 60 s twice using the specific program of the device for root canal treatment. The applied ozone concentration was 2100 ppm which resulted in 4.49 g/m^3^. However, the device has been replaced on the market in the meantime by HealOzone X4 providing an ozone concentration of 32 g/m^3^, which should be taken into consideration when interpreting the present results.

No significant differences in bacterial reduction could be demonstrated in the present study for the various investigated disinfection methods in combination of instrumentation and irrigation using 1% NaOCl highlighting the fact that a one-visit root canal treatment including instrumentation and antibacterial irrigation in combination with adjunctive disinfection methods like ozone or diode laser application is equally effective compared to a simulated two-visit endodontic treatment with application of medical dressings.

The enhanced antimicrobial effect of gaseous ozone in combination with antibacterial irrigation using NaOCl has been also demonstrated in a recent in vitro study [25]. The cited authors speculated that disintegration of the bacterial biofilm using NaOCl might result in better penetration of ozone into the bacterial biofilm and the dentinal tubules. This supports the application of gaseous ozone as an adjunctive disinfection method in combination with an antibacterial irrigation protocol and instrumentation of the root canal. However, previous studies also demonstrated complete removal of* E. faecalis* after using solely NaOCl [25, 48] questioning an additional antimicrobial effect of ozone. The present study also analyzed the long-term antimicrobial effect of the various disinfection methods with further incubation of exemplary samples of each group that demonstrated no bacterial growth immediately after treatment. Nearly 80% of ozone and laser treated samples revealed further bacterial growth whereas less than 50% of the samples with medical dressings showed further bacterial growth. These results also indicate little additional effects of the application of ozone of diode laser treatment in the present study. In this aspect, the abovementioned carry-over effect should be taken into consideration especially with the use of medical dressings. Parts of the active form of the medical dressings might have followed along with the sample into dilution series and possibly on the culture plate. A high enough concentration of the disinfectant might result in false negative results: the bacteria are not killed but might be hampered in growing because of the bacteriostatic effect. This might result in a too positive evaluation of the antibacterial methods tested [46]. In the present study, no blocking solutions for CHX like Tween 80 and alpha-lecithin or Ca(OH)_2_ like citric acid after the removal of the medical dressings have been applied and consequently the above described effects of overestimation of the antibacterial effects of the medical dressings cannot be excluded. Nevertheless, further effects of the mentioned blocking solutions can also not be excluded. Furthermore, the antibacterial effectiveness of calcium hydroxide is decreasing after application because of the reduced availability of hydroxyl ions in solution [9] and this also should minimize the carry-over effect. In addition, bacterial reduction was comparable between the application of medical dressings and irrigation using NaOCl and laser or ozone. Moreover, further bacterial growth in vivo might also be limited by complete three-dimensional root canal filling and further effects of medical dressings after incomplete removal might also occur.

In the present study, laser assisted disinfection revealed effective bacterial reduction in combination with antibacterial irrigation using 1% NaOCl and was equally effective compared to the investigated medical dressings Ca(OH)_2_ and CHX-Gel or application of gaseous ozone for planktonic and adherent cells of* E. faecalis*.

These results were corroborated by a previous study that investigated the antibacterial effect of a 908 nm diode laser (2.5 W) on* E. faecalis*.* E. faecalis* was completely eliminated using antibacterial irrigation protocols with NaOCl [49]. Another study analyzed the antibacterial efficacy of diode laser irradiation (940 nm, 3.5 W) compared to three other root canal disinfection methods: conventional irrigation, EndoActivator, and PIPS (photon-initiated photoacoustic streaming). Samples that were treated with diode laser irradiation revealed the highest antibacterial efficacy against* E. faecalis* compared to all other methods [33]. Consequently, the combination of gaseous ozone or diode laser with chemomechanical canal enlargement and NaOCl irrigation may offer an approach to single-visit root canal treatments in endodontic therapy. Nevertheless, activation of antibacterial irrigation solutions, namely, NaOCl, has not been analyzed in the present experimental approach and could also contribute to sufficient bacterial reduction in a single-visit endodontic treatment approach [10].

To date, a multiple-visit approach in endodontic therapy is still commonly performed. In a systematic review on nonsurgical single-visit versus multiple-visit endodontic treatment, Wong et al. described that up to 90% of clinical practitioners prefer a multiple-visit approach [23]. Hence, medical dressings still play an important role in endodontic therapy. Ca(OH)_2_ is the most frequently used intracanal medical dressing besides its questioned antimicrobial effectiveness [50]. The results of our investigation demonstrated significantly better antibacterial action of Ca(OH)_2_ and CHX-Gel (1%) against planktonic and adherent cells of* E. faecalis* after using it for 7 days [42] compared to disinfection solely using diode laser or ozone without an antibacterial irrigation protocol. For planktonic bacteria, Ca(OH)_2_ demonstrated a significantly higher bacterial reduction compared to CHX-Gel. No significant differences were found comparing the antibacterial effectiveness of Ca(OH)_2_ and CHX-Gel (1%) for adherent bacteria. These results were confirmed by a previous study where Ca(OH)_2_ was found to be as effective as 1% CHX in reducing* E. faecalis* at 3 and 8 days [51]. Nevertheless, intracanal remnants of Ca(OH)_2_ hinder the sealing quality of root canal filling materials, putting a risk to reinfection of the root canal system after obturation. CHX-Gel is considered to be an alternative medical dressing to Ca(OH)_2_. This topic needs to be addressed in future studies.

## 5. Conclusion

In summary, Ca(OH)_2_ was the most effective disinfection method against* E. faecalis* without any supportive irrigation protocols. Combining gaseous ozone and laser irradiation with NaOCl irrigation and instrumentation of the root canal resulted in comparable bacterial reductions of* E. faecalis* to application of medical dressings. Within the limitations of this in vitro study, it can be concluded that one-visit root canal treatment including antibacterial irrigation using NaOCl combined with instrumentation and adjunctive disinfection using ozone or laser achieved bacterial reductions of* E. faecalis* comparable to the application of medical dressings. This supports the option of sufficient bacterial reduction in a single-visit root canal treatment.

## Additional Points

One-visit root canal treatment including antibacterial irrigation using NaOCl in combination with adjunctive disinfection like ozone or laser resulted in comparable bacterial reduction of* E. faecalis* to application of medical dressings simulating a multiple-visit endodontic treatment.
